# Combined Use of Morphological and Molecular Tools to Resolve Species Mis-Identifications in the Bivalvia The Case of *Glycymeris glycymeris* and *G*. *pilosa*

**DOI:** 10.1371/journal.pone.0162059

**Published:** 2016-09-26

**Authors:** Ariadna Purroy, Tanja Šegvić-Bubić, Anna Holmes, Ivana Bušelić, Julien Thébault, Amy Featherstone, Melita Peharda

**Affiliations:** 1 Institute of Oceanography and Fisheries, Split, Croatia; 2 Department of Biodiversity and Systematic Biology, National Museum of Wales, Cardiff, UK; 3 Université de Brest, Institut Universitaire Européen de la Mer, Laboratoire des sciences de l'environnement marin (LEMAR UMR6539 CNRS/UBO/IRD/Ifremer), Plouzané, France; University of California, UNITED STATES

## Abstract

Morphological and molecular tools were combined to resolve the misidentification between *Glycymeris glycymeris* and *Glycymeris pilosa* from Atlantic and Mediterranean populations. The ambiguous literature on the taxonomic status of these species requires this confirmation as a baseline to studies on their ecology and sclerochronology. We used classical and landmark-based morphometric approaches and performed bivariate and multivariate analyses to test for shell character interactions at the individual and population level. Both approaches generated complementary information. The former showed the shell width to length ratio and the valve asymmetry to be the main discriminant characters between Atlantic and Mediterranean populations. Additionally, the external microsculpture of additional and finer secondary ribs in *G*. *glycymeris* discriminates it from *G*. *pilosa*. Likewise, landmark-based geometric morphometrics revealed a stronger opisthogyrate beak and prosodetic ligament in *G*. *pilosa* than *G*. *glycymeris*. Our Bayesian and maximum likelihood phylogenetic analyses based on COI and ITS2 genes identified that *G*. *glycymeris* and *G*. *pilosa* form two separate monophyletic clades with mean interspecific divergence of 11% and 0.9% for COI and ITS2, respectively. The congruent patterns of morphometric analysis together with mitochondrial and nuclear phylogenetic reconstructions indicated the separation of the two coexisting species. The intraspecific divergence occurred during the Eocene and accelerated during the late Pliocene and Pleistocene. *Glycymeris pilosa* showed a high level of genetic diversity, appearing as a more robust species whose tolerance of environmental conditions allowed its expansion throughout the Mediterranean.

## Introduction

Mollusc shells have been recognized as useful archives of environmental data spanning in time from several years to millennia [1–3]. Based on their widespread distribution, from polar to tropical regions, and from freshwater to saltwater ecosystems, bivalve shells can provide valuable information pertinent to the reconstruction of environmental variations [4]. Sclerochronological analysis of bivalve shells includes the investigation of interannual variations in growth increment widths as well as variations in geochemical composition and these studies have developed rapidly over the past decade [5]. The longest-lived bivalve, *Arctica islandica* (Linnaeus, 1767), has been extensively used for sclerochronology in the North Atlantic and the maximum longevity of this species has been estimated at 507 years [1–2,6–7]. The potential for another species to act as a paleoenvironmental archive, *Glycymeris glycymeris* (Linnaeus, 1758), has been recently tested in the Irish Sea [8], NW France [9] and NW Scotland [3]. The expansion of sclerochronological studies into the Mediterranean requires identification of bivalve species that could be used as paleoenvironmental archives. Studies on species of *Glycymeris* are especially important since there are no comparable long-lived bivalves in the Mediterranean. In addition, species of glycymerids are locally abundant both alive as well as in in the fossil record [10–13]. To date, studies on *Glycymeris* in this region are few and only one growth chronology, that for *G*. *bimaculata* (Poli, 1795), has been published [14].

The coexistence of two other species, *Glycymeris glycymeris* and *Glycymeris pilosa* (Linnaeus, 1767) hamper their use as proxies because they have been historically misidentified. An uncertain distribution and frequency of *G*. *glycymeris* in the Mediterranean has been recognized due to confusions with another species of the same genus—*G*. *pilosa* [15]. Other studies considered them as two forms of the same species because of their heavy and globose shapes (see description in [16]). There is no doubt about the presence of *G*. *glycymeris* in the North Atlantic, however, its presence in the Mediterranean has been poorly documented and there is a lack of studies on living individuals. Likewise, there are discrepancies whether or not *G*. *pilosa* (but treated as *G*. *glycymeris*) has been historically collected from the Eastern Atlantic. Some systematic papers have classified *G*. *pilosa* as a variety of *G*. *glycymeris* [17–18]. According to the Check List of European Marine Mollusca [19], both species are still considered as synonyms. Abundant populations of *G*. *pilosa* can be found in the Adriatic Sea. They have, however, been mistakenly identified as *G*. *glycymeris* in earlier studies [12]. Only during the last decade, have taxonomists started to identify them as two different species based on morphological characters [16,20]. The World Register of Marine Species [21], aiming to resolve critical issues of nomenclature, also considers them to be distinct species. For this reason, clarifying the erroneous consideration of *G*. *glycymeris* and *G*. *pilosa* as synonymous is essential for any further studies on sclerochronology and ecology.

According to the literature, *Glycymeris glycymeris* is distributed in the Northeast Atlantic (the Hebrides, Faroe Islands, Norway, North Sea, English Channel and Bay of Biscay), the Azores, Canaries and Madeira Archipelago’s and the Mediterranean. Likewise, *Glycymeris pilosa* has been recorded from Western Sahara, Mauritania, Madeira and the Canaries Archipelago’s and the Mediterranean Sea (up to Israel) [15–16,18,20,22].

Glycymerids are descendants of the ancient Arcoidea, a distinct lineage that was established early in the radiation of the Bivalvia [23–24]. Since its appearance during the Cretaceous (~130 Ma), fossil individuals seem to occupy similar ecological ranges to the present species in the North Atlantic, which could be explained by generic conservatism [23]. They are not as specialized anatomically as other bivalves; instead, they have adapted to physically harsh environments evidencing their role as functional generalists [23]. The high degree of conservatism expressed by the genus *Glycymeris*, not only in terms of their morphology [23] but also reflected in shell ultrastructure [11], and a slow adaptation to different environments have added difficulties to the process of distinguishing species. Often, the variation between species is determined by differences in size and external sculpture. Such changes may have been triggered by adaptations to different environments and can be detected using both morphological and molecular phylogenetic tools (i.e. molecular clock) [25]. Species of *Glycymeris* have a very robust shell and prefer gravel to hard substrata under strong current regimes rather than in fine sediments and calm waters [26]. They are shallow burrowers with mainly nocturnal behaviours [23]. Earlier studies quantitatively measured and interrelated a wide range of glycymerids shell characters [23]. Aiming to classify species of *Glycymeris*, Goud & Gulden [20] selected certain distinctive morphological traits to distinguish between *G*. *glycymeris* from *G*. *pilosa*. Some of these traits coincided with those espoused in Thomas [23] but they also introduced others including shell microsculpture measurements of primary and secondary rib counts, which, it transpired, were species-uniform and distinct. The importance in modern taxonomy of DNA analyses contrasts with morphological studies in biodiversity research [27]. The exclusive reliance on one or other method may, however, fail to detect variations. Comprehensive studies including morphometrics and molecular analyses may, therefore, provide a more accurate approach to species discrimination. The genetic analyses of bivalve species identifications have been used recently to correct mislabeling [28–29]. The identification of molecular markers (i.e. mitochondrial and nuclear genes) has also been used to track genetic diversity and population structure [24,30–31]. Other methods such as the molecular clock have been used to describe phylogenetic relationships and evolutionary histories [32–34].

The aim of this study was to resolve the historical misidentification of *G*. *pilosa* in the Mediterranean and corroborate its separation from *G*. *glycymeris* as distinct species by combining morphometric and genetic analyses. This has been achieved by measuring several characters, following classical and landmark-based approaches, and through mitochondrial (COI) and nuclear (ITS2) markers.

## Materials and Methods

### Study area

Atlantic specimens came from the Isle of Man (54° 26’ 54.49” N, 4° 20’ 21.73” W) issued by the Department of Environment, Food and Agriculture (authorization n°SF204.011/2015), and from the Bay of Brest (48°20’ 29.88” N, 4°30’ 45.66” W) issued by the Directorate of Maritime Affairs (authorization n°101/2014), hereafter referred to as UK and France groups, respectively. Mediterranean samples were collected in two locations along the eastern Adriatic coast in Croatia: Pag Bay (44° 27’42” N, 15°01’36”E) and Pašman Channel (43°56’49”N, 15°23’18” E) issued by the Ministry of Science, Education and Sport (authorization n°533-19-14-0008/2014 and 533-19-14-0006/2015), hereafter referred to as Pag and Pašman sample groups, respectively. Unfortunately we were unable to collect specimens of both species neither in the Atlantic nor the Mediterranean.

**Fig 1:**
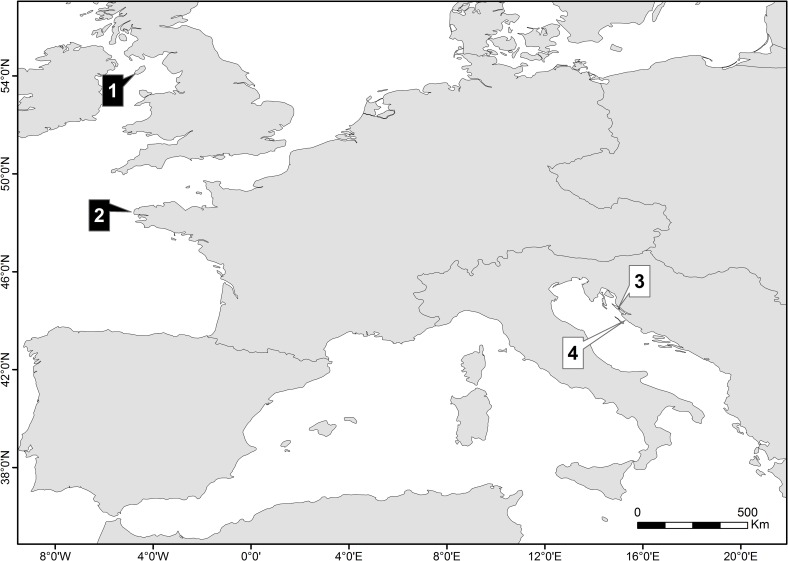
Collection sites along the Northeast Atlantic and Mediterranean Sea. Specimens identified as *Glycymeris glycymeris* (black fill) and *Glycymeris pilosa* (white fill) came from: (1) Isle of Man (UK), (2) Bay of Brest (France), (3) Pag Bay (Croatia), (4) Pašman Channel (Croatia).

A total of 107 shells classified as either *G*. *glycymeris* or *G*. *pilosa* were live collected. Samples from UK and France represented two groups of *G*. *glycymeris* (17 and 30 specimens, respectively), whereas two groups of *G*. *pilosa* came from Pag and Pašman (30 specimens from each location) (Fig 1). Due to logistical constraints (limited to one sampling cruise) and physical damage during handling, sample size of the UK population was smaller. Monthly sampling was performed at other sites ensuring enough samples in good condition. All morphometric measurements were conducted on the right valve (identified according to the position of the beaks, turned towards the posterior end of the valves; [16]). Samples from collections in the National Museum of Wales, Cardiff were also used for corroboration of measurements. All material examined with accession number beginning NMW.Z is held in the collections at the NMW and is available for institutional loan on request. The remaining material is held at the Institute of Oceanography and Fisheries (IOF), Split (S1 Table).

### Morphometric analysis

Classic and novel morphometric analyses were chosen based on the bidimensional and tridimensional approaches of each method, to explore the range of morphological variation. Due to the absence of sexual dimorphism in the shell, males and females were treated indistinguishably [23]. To identify shell morphometric relationships among populations we conducted bivariate and multivariate analysis.

#### Classical morphometric analysis

**Fig 2:**
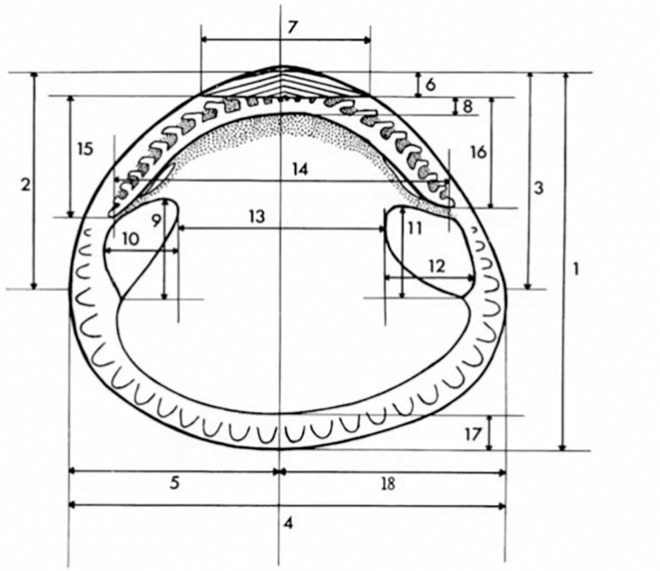
Classical morphometrics measurements. Height of shell (measured from umbo)(1), height of anterior extremity (2), height of posterior extremity (3), length of shell (4), anterior length (5), posterior length (18), asymmetry (5/18), height of ligamental area (6), length of ligamental area (7), median height of hinge plate (directly below umbo)(8), height of ligamental area / height of hinge plate (6/8), height of anterior tooth row (15), height of posterior tooth row (16), distance between last anterior and posterior teeth (14), height of anterior adductor scar (9), length of anterior adductor scar (10), area of anterior adductor scar (9*10/2), height of posterior adductor scar (11), length of posterior adductor scar (12), area of posterior adductor scar (11*12/2), distance between inner margins of adductor scars (13), adductor moment = sum of adductor scar areas*mean distance from hinge axis, height of crenulated extra-pallial margin (17) and the width (W). Measurements taken for each morphometric character adopted from Thomas (1975) with permission from the Palaeontological Association. Right valve of a Glycymerid.

The basis for the selected morphological data came from Thomas [23]. This study described the evolutionary conservatism of the Glycymerididae family by looking at the interrelationships of shell characters (Fig 2). The characters were measured with a digital caliper (accuracy of ± 0.02 mm). Additional morphological characters were included based on sculptural traits (e.g. rib counts). This step was introduced in our analyses because Goud & Gulden [20] counted the number of secondary ribs within a primary rib to separate *Glycymeris* species; however, we were unable to consistently see the primary ribs clearly enough to be able to use this as a unit of measurement for the secondary ribs. Instead, a fixed length of shell (5 mm) was counted for secondary ribs. Externally, measuring 30 mm from the beak to the middle external of the valve, a marker measuring 5 mm was temporarily adhered at a right angle to the secondary ribs. In this way a consistent distance of shell was counted for secondary ribs. A tiny sticker marked with a 5 mm ruler curved with the shell to get an accurate count for each specimen. This method also ensured that if secondary ribs increased with age and hence closer to the ventral part of the shell, then shells of different sizes/ages could still be used in the count, provided they were not smaller than 30 mm in height.

To reduce redundancy in the Principal Component Analysis (PCA), we used Pearson correlation coefficients (through linear regression analyses) for data exploration. Those characters from the morphometric dataset that expressed more significant linear relationships among the populations were computed as ratios for a size free shape multivariate analyses. A PCA was performed after data standardization on: shell width to shell length (W/L), shell length to shell height (L/H), asymmetry to shell height (A/H), height of ligament to shell height (HL/H), height of ligamental area to shell height (LA/H), length of ligamental area to shell length (LL/L), area to shell size ratio of both the anterior and posterior adductor muscles (AM/S & PM/S), height of the crenulated extra-pallial margin to shell height (M/H) and the log of the adductor moment (logAM) to find those variables contributing the most to the individual variability. Due to the wide range of variables at different scales a correlation matrix was used in the PCA. A Linear Discriminant Analysis was sought to discriminate differences among populations. Statistical analyses were carried out using the open software PAST v3.0 [35].

#### Landmark-based geometric morphometrics (GM)

**Fig 3:**
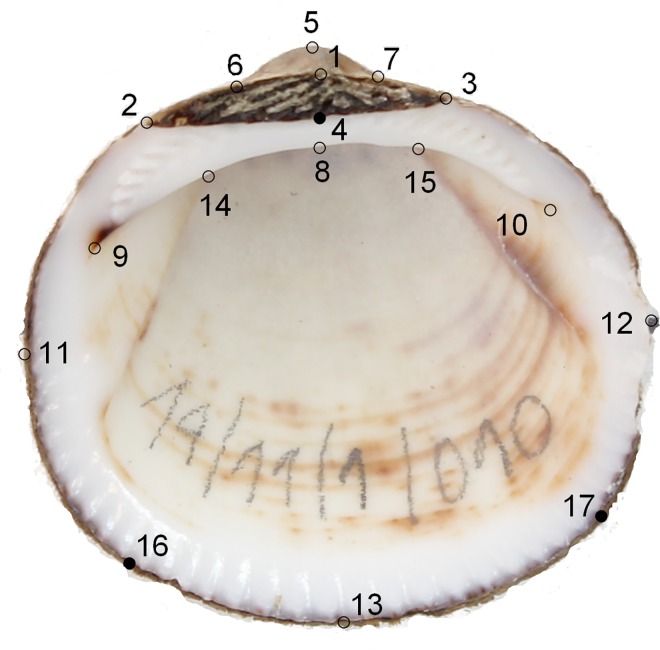
Geometric morphometrics landmarks. This image represents a right valve of *Glycymeris glycymeris* with the position of 12 landmarks (open dots) and 3 semi-landmarks (fill dots).

The GM analyses implemented in software MorphoJ [36] were used to quantify shape variation from 60 images of *Glycymeris* sp. from different localities (15 samples from each group). For all specimens, the inner side of the right valve was photographed and digitized with the software TPS dig2 [37]. From these images 12 landmarks and 2 semi-landmarks were defined (Fig 3). Landmarks were subjected to a standard generalized Procrustes alignment to remove differences between specimens based on scale, rotation, and location. A PCA was also applied to the Procrustes coordinates to explore variation in the shape components. Multivariate regression of shape with centroid size as the independent variable was computed to assess intrapopulation allometry. Permutation test with 10,000 rounds was used to evaluate the independence between the shape and size variables. Finally, a canonical variate analysis (CVA) was performed to find out which shell shape features best discriminated between species.

### Sampling and DNA extraction

Based on the preliminary morphological determination, 15 specimens from each location were allocated for DNA sequencing. In addition, two specimens of *G*. *nummaria* (Adriatic origin) and two of *G*. *bimaculata* (from Balearic and Adriatic Seas) were sampled for phylogenetic analysis. Adductor muscle samples were stored in 96% ethanol. Genomic DNA was extracted from 30–50 mg of tissue using the DNeasy Tissue Kit (Qiagen Inc.) following the manufacturer’s protocol. Two molecular markers were analyzed; internal transcribed spacer 2 (ITS2) rDNA was amplified according to Oliverio & Mariottini [38] while a partial fragment of the mitochondrial cytochrome c oxidase subunit I gene (COI) was amplified following Folmer et al. [39]. Products sequencing were performed by Macrogen Inc. (Seul, Korea) on an ABI 3730 automatic sequencer.

From GenBank database, 5 species of Arcida (*Arca ventricosa*, Accession number AB076935.1; *Tegillarca granosa*, HQ896817.1; *Cucullaea labiata*, AB050892.1; *Cosa waikikia*, AB084107.1; *G*. *glycymeris*, KC429093.1) were additionally used as ingroup samples for COI reconstruction while two pteriomorph species from different orders (*Isognomon legume*n, AB076950.1; *Spondylus gaederopus*, JF496776.1) and the protobranch *Nucula atacellana* (KJ950273.1) were chosen as outgroups (see [34]). To fulfill ITS2 data set, *G*. *glycymeris* (FN667988.1) originating from Tuscan Archipelago, Mediterranean Sea [32] were used to accommodate intra family analysis.

Sequences were screened using BLAST searches (ncbi.nlm.nih.gov), trimmed and aligned in ClustalW 2.1 [40]. All new haplotypes have been deposited in GenBank database under Accession numbers: KX785175-KX785221.

### Genetic diversity and phylogenetic analyses

Molecular diversity was measured using Dnasp 5.10 [41] calculating the number of haplotypes (H), polymorphic sites (S), haplotype and nucleotide diversity. Intra- and interspecific distance was calculated in MEGA6 [42]. The demographic histories for *G*. *glycymeris* and *G*. *pilosa* were tested using Tajima’s D [43] and Fu’s FST [44], where negative values indicated population expansion or historical bottleneck [45]. The significance of tests were assessed using 10,000 samples simulated under a model of constant population size in Arlequin 3.5.1.2 [46].

Phylogenetic reconstructions were estimated using maximum likelihood (ML) and Bayesian (BI) analysis on COI and ITS2 datasets. jModelTest [47] was used to determine the most suitable model of sequence evolution under the Akaike information criterion (AIC). The general time reversible with the gamma distribution shape parameter (GTR+G) model was chosen for COI gene and Jukes–Cantor [48] for ITS2 gene. A BI tree was constructed using MrBayes 2.0.6 [49] as implemented in Geneious (v. 2.0.3) running at least two independent Monte Carlo Markov Chain (MCMC) analyses with 2,200,000 generations sampled every 400 generations, with a 200,000 tree burn-in. Maximum likelihood trees were constructed using the PhyML [50] plug-in of Geneious with the BEST topology search option and 1000 bootstrap replicates.

### Divergence time estimates

Divergence times of *G*. *glycymeris* and *G*. *pilosa* were estimated using BEAST 1.8.1 [51] as implemented on the CIPRES web portal [52], on the COI dataset. In respect to Combosch & Giribet [34], three fossil-based calibration points were used: (i) the root age of Bivalvia between 520.5 and 530 Ma, (ii) the age of Arcida at 478.6 (±5) Ma based on *Glyptarca serrata* [53], and (iii) the age of Glycymerididae at 167.7 (±5) Ma based on *Trigonarca tumida* [54]. Analyses were performed under relaxed uncorrelated lognormal clocks and a constant size coalescent population model. These priors were selected according to the Bayes factors from TRACER 1.6 [55] calculated to compare models, although all tested models including strict or relaxed molecular clock combined to exponential growth coalescent population model or Yule process speciation model produced similar divergence time estimates. Four independent runs with MCMC chain length of 5×10^7^ were conducted, sampling every 5×10^3^ generations. Convergence diagnostics were checked using [55] and phylogenetic trees were summarized in a target tree by the Tree Annotator program included in the BEAST package by choosing the tree with the maximum clade credibility after a 50% burn-in.

### DNA-based species delimitation

For single marker species delimitation (COI), coalescent tree-based methods as the generalized mixed Yule-coalescent model (GMYC; see [56]) and the Poisson tree process model (bPTP; see [57]) were applied in order to identify the number of phylospecies. The GMYC analysis was conducted using R Statistical Platform [58], with the use of splits package. The input for the GMYC was an ultrametric single locus gene tree obtained with BEAST [51]. The coalescent tree-based bPTP method was performed using the web interface available at http://species.h-its.org/ptp/ on the Bayesian majority-rule consensus non-ultrametric tree as input.

## Results

### Morphometric analysis

**Fig 4:**
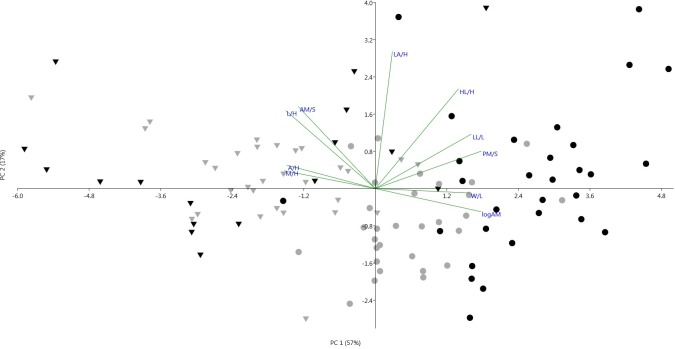
Biplot of the mean scores on the first two principal components based on 10 morphological character ratios. Symbols were assigned to each population: *Glycymeris glycymeris* (black inverted triangle, UK; grey inverted triangle, France) and *Glycymeris pilosa* (black circle, Pag; grey circle, Pašman).

Classical morphometric variables showed significant differences among populations (MANOVA, Wilk’s Lambda test, F = 17.43, P < 0.001). As a result of bivariate analysis among all the 24 measured characters, the dataset to carry out PCA was reduced to those areas representing the umbo region, adductor muscles and shell dimensions contributing in a total of 10 ratios (W/L, L/H, A/H, HL/H, LA/H, LL/L, AM/S, PM/S, M/H, logAM). Linear regression analyses indicated the shell width to be highly correlated with the shell length in populations of *G*. *pilosa* (r > 0.94, p < 0.001, with a R^2^> 0.89) and somewhat less for *G*. *glycymeris* (r > 0.77, p < 0.001, with a R^2^ = 0.57). Geographically, Pag presented the most dispersed data. The height of the ligament to shell height showed a high correlation in all populations except Pašman (R^2^ > 0.66, p < 0.001 and R^2^ = 0.32, p < 0.05, respectively). Contrary, the shell length to shell height (R^2^ > 0.85, p < 0.001), the length of the ligamental area to shell height (R^2^ > 0.59, p < 0.001), anterior adductor muscle area to shell height (R^2^ > 0.61, p < 0.001) and the log of the adductor muscle (R^2^ > 0.82, p < 0.001) did not distinguish among populations. Asymmetry, marked major differences between Atlantic (R^2^ > 0.81, p < 0.001,) and Mediterranean (R^2^ < 0.08, p > 0.05) populations, where the later was the most asymmetric. UK appeared as the most symmetric population (R^2^ = 0.25, p > 0.05) and Pašman as the most asymmetric (R^2^ = 0.06, p > 0.05). The length of the ligament indicated a greater correlation to shell length for shells from Brest, UK and Pag (R^2^ > 0.86, p < 0.001), while data were a bit more dispersed for Pašman (R^2^ < 0.64, p < 0.001). The margin height (R^2^ > 0.51, p < 0.001) and the posterior adductor muscle area (R^2^ > 0.63, p < 0.001) showed a high correlation to shell height for Brest, UK and Pašman populations. Population in Pag appeared less correlated for these features (R^2^ = 0.16, p < 0.05 and R^2^ = 0.37, p < 0.001, respectively). The first Principal Component (PC 1) explained 57.4% of the variation (eigenvalue 5.74), weights on W/L, A/H, L/H, LL/L, PM/S, M/H and the logAM were highly positively. The weightings for PC 2 (eigenvalue 1.67) indicated LA/H and HL/L to be the most important, explaining a 16.7% of the variation (Fig 4).

The first linear discriminant (LD 1) explained the majority of the total variance (90.96%) (S1 Fig). The Confusion matrix in LDA (Table 1) showed that misplacements of individuals in populations of the same species represented 6.6%, 17.6%, 3.3% and 26.6% for Brest, UK, Pag and Pašman populations, respectively. The intraspecies variability was higher for *G*. *pilosa*.

**Table 1:** LDA Confusion matrix on the four populations of *Glycymeris*.

	Brest	UK	Pag	Pašman	Total
Brest	28	2	0	0	30
UK	3	14	0	0	17
Pag	0	0	29	1	30
Pašman	0	0	8	22	30
Total	31	16	37	23	107

**Fig 5:**
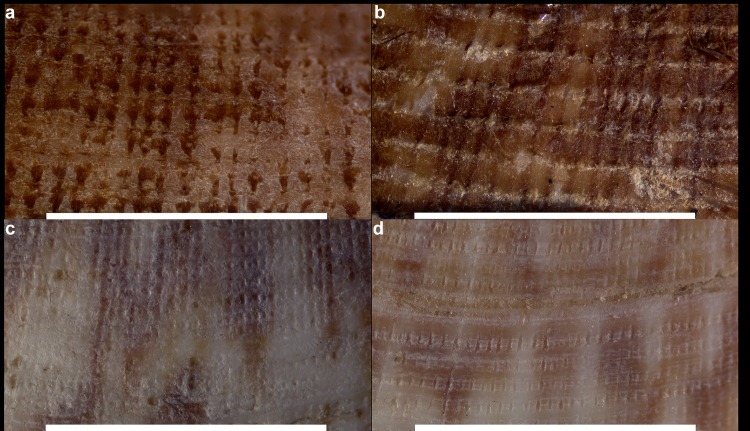
Detail of the microsculpture and rib count measurements of *Glycymeris pilosa* and *Glycymeris glycymeris*. The white line is used as a scale of 5mm. Measured populations correspond to (a) Pašman, (b) Pag, (c) UK and (d) Brest.

Concerning the microsculpture, *G*. *pilosa* had a more robust, rugged sculpture compared to the finer reticulate sculpture of *G*. *glycymeris* (Fig 5). A total of five specimens each of Pašman, Pag, Isle of Man and Brest samples were used to determine if the secondary ribs differed between shells collected at different locations (Table 2).

**Table 2:** Secondary rib counts counted on the microsculpture in all four *Glycymeris* populations.

Location	Specimen 1	Specimen 2	Specimen 3	Specimen 4	Specimen 5
Pag Bay	12	12	15	12	15
Pašman Channel	14	14	10	11	12
Isle of Man	26	22	25	23	25
Bay of Brest	28	25	26	25	26

Pag Bay and Pašman Channel, the two populations of *G*. *pilosa*, fall between 10–15 secondary ribs per 5 mm. Isle of Man and Brest, the two populations of *G*. *glycymeris*, fall between 22–28 per 5 mm. This is consistent with other specimens of *Glycymeris* from other locations from the collections of the National Museum of Wales and other populations of *G*. *pilosa* collected from Istria and Cetina River. Additional material from the museum collection was included in the measurements (S1 Table).

**Fig 6:**
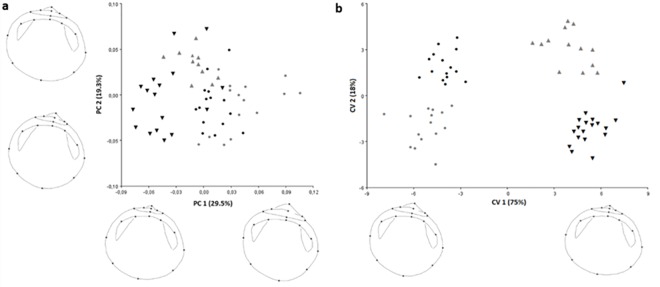
Plot of the principal components (PCs) based on Procrustes distances (a). Shape changes associated with the PCs are shown as extreme shell shapes representing the positive and negative end of each axis. Percentages of explained variance for each axis are in parentheses. Plot of the canonical variate analysis (CVs) of overall shell shape variation along the first 2 canonical axes (b). Wrapped outline drawings show shape changes associated with variation along first axis. *Glycymeris glycymeris* (black inverted triangle, UK; grey inverted triangle, France) and *Glycymeris pilosa* (black circle, Pag; grey circle, Pašman). Percentages of explained variance for each axis are in parentheses.

Following the landmark-based geometric morphometrics, growth allometry of the *Glycymeris* sp. was observed and accounted for 14% of the total amount of shape variation. The resulting PCA revealed that the first two components PC 1 and PC 2 (eigenvalues 0.0015 and 0.0010) explained 29.5% and 19.3% of the total variability among the landmarks, on all analyzed specimens (Fig 6). At the primary axis of variation (PC 1), segregation by the species (*G*. *glycymeris* vs. *G*. *pilosa*) was observed, and it mainly described changes in the umbo region and enlargement of the posterior-ventral axis of the shell. The second PC axis was related to the development of the ligament area and elongation of the anterior region. The shell shape variations among species from different localities were successfully discriminated using CVA (Fig 6). All groups differed significantly between each other as revealed by permutation testing of Procrustes distances. The morphological differentiation measured by Procrustes distance was similarly large between the *G*. *glycymeris* from both sampling locations and *G*. *pilosa* from Pašman (0.09) or Pag (0.06), respectively. The population distances within species (*G*. *glycymeris* from UK vs. France, *G*. *pilosa* from Pag vs. Pašman) were similar and considerably smaller (0.05–0.04). The first canonical axis (CV 1) explained the majority of the total variance (75%). Depicting the between species changes along discriminant functions by warped outline drawings revealed that shape was altered most in umbo and upper posterior-anterior region (Fig 6).

**Fig 7:**
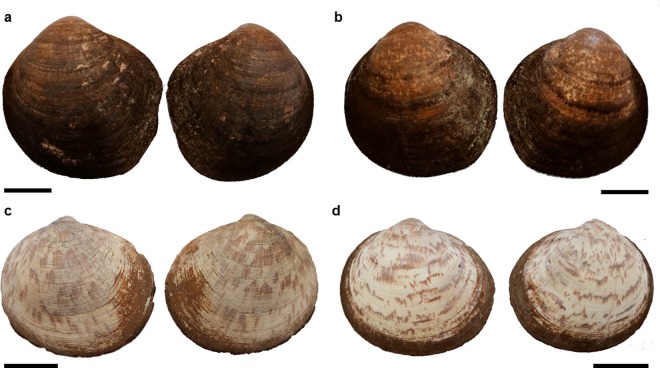
Left and right valves of *Glycymeris pilosa* from Pašman (a) and Pag (b) and *Glycymeris glycymeris* from UK (c) and Brest (d) populations. Scale bar 2 cm.

Complementary, images of both shell valves are shown in Fig 7, represented by one specimen from each population.

### Molecular analysis

Fragments of 669 and 299 bp were obtained for COI and ITS2 genes, respectively. A total of 16 COI haplotypes and three ITS2 haplotypes were identified among *G*. *glycymeris* dataset. The COI gene contained 15 variable sites, whereas the ITS2 gene contained only two variable sites. For the *G*. *pilosa* dataset, a total of 32 variable sites and 19 haplotypes were identified for COI gene while the ITS2 gene contained only one variable site and two haplotypes. Genetic diversity indices for each gene and species are summarized in S2 Table, indicating that COI displayed quite higher values of both haplotype and nucleotide diversities (h = 0.96; π = 0.056) in comparison to the nuclear gene (h = 0.62; π = 0.005). For both genes, the most common haplotypes of *G*. *glycymeris* and *G*. *pilosa* were presented in both sampling locations (S3 Table). COI interspecific divergence accounted for 11% and for ITS2 was 0.9%, respectively. Mean intraspecific divergence in the *G*. *glycymeris* dataset were 0.4% (COI) and 0.04% (ITS2), while in the *G*. *pilosa* were 1.1% (COI) and 0.1% (ITS2), respectively.

Neutrality tests applied to search demographic species pattern revealed significant negative values for Tajima’s D and Fu’s F_ST_ for both *G*. *glycymeris* and *G*. *pilosa*, indicating a recent expansion of mtDNA haplotypes (S3 Table).

**Fig 8:**
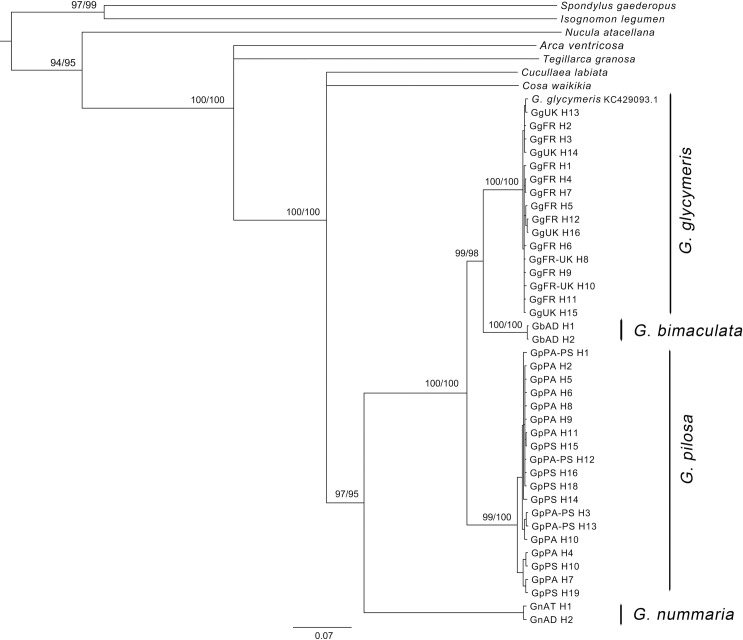
Bayesian posterior probabilities and bootstrap support for MrBayes and Maximum Likelihood analyses. Illustration of tree topology based on COI haplotypes of *G*. *glycymeris* (Gg), *G*. *pilosa* (Gp), *G*. *nummaria* (Gn), *G*. *bimaculata* (Gb) and outgroups. Posterior probabilities followed by bootstrap values are included at the nodes. The origin of the haplotypes (H) is indicated as follow: UK, United Kingdom; FR, France; AD, Adriatic Sea; AT, Atlantic Sea; PA, Pag; PS, Pašman.

For COI gene, the dated topology (BEAST), Bayesian and ML analysis gave congruent results. Phylogenetic reconstructions distinguished four strongly supported species of Glycymerididae (bootstrap support 97% to 99%, posterior probabilities 0.95–0.99) in the ingroup (Fig 8). Also, the analyses of COI data supported close relationship between *G*. *glycymeris* and *G*. *bimaculata*, having *G*. *pilosa* species as a sister clade. By contrast, deep genealogical divergence among *G*. *nummaria* and other Glycymeris species were recorded. The ITS2 tree (S2 Fig) was much less resolved, with three well-supported groups recognized, among which *G*. *glycymeris* and *G*. *bimaculata* were grouped into one clade. The *G*. *glycymeris* specimen (FN667988.1) originating from Tuscan Archipelago, Mediterranean Sea was assigned in G. *pilosa* clade, suggesting species mislabeling in the GenBank database.

The divergence time for *G*. *nummaria* clade was estimated to occur at around 142 Ma (95% highest posterior density interval [HPD] 132.5–151.5), while divergence of *G*. *pilosa* clade at one side and *G*. *glycymeris* and *G*. *bimaculata* clades on the other, started to occur at around 46 Ma (95% HPD 20.9–80.1). The divergence time of *G*. *glycymeris* and *G*. *bimaculata* clades was dated around 25.3 Ma (95% HPD 9.5–45.3). Intraspecific haplotype divergence of *G*. *pilosa* started to occur 7 Ma (95% HPD 2.7–11.8) with the major haplotype diversification observed during late Pliocene and Pleistocene (0.4–3.0 Ma). Intraspecific divergence in the *G*. *glycymeris* clade also predates the Pleistocene (0.4–2.5 Ma) (S3 Fig).

Both species delimitation analyses performed by implementing the coalescent tree-based approach (GMYC and bPTP) produced similar results and provided strong support for the *a priori* defined morphospecies. The GMYC model delimited 11 species (4 *Glycymeris* and 7 outgroup species) with a confidence interval of 9 to 13, and exhibited a significantly better likelihood than the null model (ML GMYC = 648.6, lnL NULL = 656.5; p-value < 0.001), pointing that a boundary between and within species was identified. The bPTP method identified on average 12.5 species (estimated number of species between 9 and 17) by simple heuristic search, resulting in Bayesian support values above 0.8.

## Discussion

The combination of two morphometric analyses (classical and GM approaches) and the use of molecular tools allowed the identification of *G*. *pilosa*, avoiding confusion with *G*. *glycymeris* and selecting the main features that distinguish them. The tridimensional nature of using the classical method alongside the landmark-based approach allowed measurement of width to length ratio (W/L), which became one of the main discriminant traits, while at the same time pointing out the variations in the umbo region and the ligamental area, which otherwise would have been hidden. Based on our results a set of 7 characters explained nearly 50% of the individual variation that distinguished *G*. *glycymeris* from *G*. *pilosa* (Fig 4). The bivariate analyses allowed identifying the morphological meaning of these characters indicating that Mediterranean populations presented a more globose and posterior-ventral elongated shell. UK specimens were the most symmetric population, whereas Pašman was the most asymmetric. Pašman specimens also showed an allometric growth in other characters such as the height of the ligament to shell height ratio (HL/H) or the length of the ligament to shell length (LL/L). Allometry has a greater effect in larger individuals, usually together with an elongation of the posterior-ventral margin [23]. Accordingly, our results were consistent with these observations since individuals from Pašman, were also the largest specimens. However, glycymerids present a very high individual variation in asymmetry, which might be site or size specific [23]; thus, this character (A/H) should be considered along with others before assuming it distinguishes between species. In the PCA analyses, the ligamental length to shell length ratio (LL/L), the length to height ratio (L/H) and the logAM strongly separated the Mediterranean and the Atlantic populations; however, this separation contrasted with the high correlation of these three variables observed in all populations; thus, they should not be used alone for species distinction. The trend for the length to height ratio was also observed in other studies [20]. The dimyarian condition of *Glycymeris*, characterized by the presence of two adductor muscles, was not a distinctive character for most populations neither in the anterior and posterior adductor muscle dimensions nor the log of the adductor moment. Our results indicated muscle sizes to be constant through ontogeny based on the differences in size as described in other studies (see [23]). LDA confirmed differences among populations and species and also showed the magnitude of the intraspecific variation.

The GM analyses showed that the beaks appeared strongly opisthogyrate, that is, pointing towards the posterior margin and this feature was more evident in *G*. *pilosa* than in *G*. *glycymeris*, as previously observed [20,16]. A prosodetic condition of the ligament (ligament lying in front of the beak) was more marked in *G*. *pilosa* rather than *G*. *glycymeris* (amphidetic to slightly prosodetic), a feature that wasn’t contemplated in the classical approach but it appeared as an outstanding variable in PC 2 (Fig 6). This secondary component of variation is explained by the measurement of adult individuals; prosodetic ligament is a character mostly manifested in juveniles of *G*. *pilosa*, thus, it could be a weak distinctive character [16]. Our results corroborated this observation and do not support the statement that the ligament condition is a clear feature to distinguish between species, at least, if age is not taken into consideration [18,23].

The use of secondary ribs has been previously used to differentiate between *Glycymeris* sp. [20,59]. These differences were corroborated in our detailed observation on microsculpture, which allowed distinguishing between species; *G*. *glycymeris* presented a finer sculpture and thus, a higher number of ribs, than *G*. *pilosa*. Additionally, information from live collected specimens can be useful for a reliable identification of species. Associated with the microsculpture is the distribution of the periostracum, a very distinctive trait from live collected specimens, with a greater length and density of periostracal hairs and a velvety appearance in *G*. *pilosa* and finer and less hairy in *G*. *glycymeris*. Of course, periostracum is not preserved in fossils. A complementary morphological feature in living specimens is their coloration. Whereas *G*. *glycymeris* tend to present reddish zigzag bands over a light yellow to dark brown bottom on the outer shell surface, *G*. *pilosa* varies from light to dark brown background where zigzag bands are hard to identify [18] (A.Purroy pers. obs.). The interior of the valves is normally paler in *G*. *glycymeris* whereas *G*. *pilosa* presents a partial or complete dark brown to violet coloration blotch, covering at least the posterior ventral area. This coloration studied in *G*. *nummaria* and *G*. *bimaculata* was reported by Crnčević [60] and Eterović [61] to be caused by endolith activity.

Aiming to enable the application of our morphometric results, the following summarized statements can be of assistance when discriminating between both species. Globosity (W/L) and posterior-ventral elongation—measured as asymmetry—(A/H) were more evident in *G*. *pilosa* although they should not be stand-alone characters for species discrimination since allometry and individual variation may have a great effect. Measurements on the length and height of the ligamental area with respect to shell length (LL/L) and height (LA/H) on the first, and to shell height (HL/H) on the latter were not very indicative of differences, except in the population with largest specimens (Pašman) where HL/H and LL/L were less correlated. Further, observations on the ligamental condition showed to be slightly prosodetic in *G*. *pilosa*, although it is a character mostly manifested in juveniles thus a weak distinctive trait by itself. The strongly opisthogyrate beaks were more evident in *G*. *pilosa*. In contrast, both adductor muscle dimensions were quite constant through ontogeny and they would not be distinctive. Complementary, when considering both living and well-preserved fossils, looking at the secondary ribs present on the microstructure appeared to be a very reliable one, showing a higher number and a finer sculpture in *G*. *glycymeris*. In addition, on the periostracum, *G*. *pilosa* presents a greater length and density of periostracal hairs, quite distinguishable in live-collected specimens. Also, on the outer shell surface, the characteristic reddish zigzag bands over yellow to dark brown bottom from glycymerids, which was observed in *G*. *glycymeris*, were much lighter or absent in *G*. *pilosa*. Further, the inner valve presents a brown to violet coloration blotch in most specimens whereas is paler in *G*. *glycymeris*. Altogether, the main discriminant traits when measured along were the globosity, and the umbo and ligamental areas. Clearly, a well-preserved shell increases the confidence in discriminating between species.

The congruent patterns of mitochondrial and nuclear phylogenetic reconstructions indicated the separation of the two co-existing species, *G*. *glycymeris* and *G*. *pilosa*, with a 100% bootstrap support and 99% posterior probability. Such a conclusion is accompanied with the high divergence rate (11%) observed between two mtDNA lineages. Also, it has been seen that the COI marker is more informative for the phylogeny of both higher-level and closely related *Glycymeris* species, whereas the ITS2 showed less resolving reconstruction for closely related species such as *G*. *glycymeris* and *G*. *bimaculata*. Due to the slower evolution rate of nuclear genes, a considerable structure can appear in mitochondrial genes before concurrent changes occur in nDNA [62]. Interestingly, the COI phylogenetic reconstruction revealed that *G*. *bimaculata* from Mediterranean Sea clustered together with G. *glycymeris* from Atlantic Sea, with a later divergence in comparison to *G*. *pilosa*. This interesting evolutionary scenario, not supported by morphospecies characteristics in our study, should be investigated in detail to confirm *G*. *bimaculata* species-specific distinctness.

Both *G*. *glycymeris* and *G*. *pilosa* showed absence of phylogenetic structure, i.e. the most frequently observed haplotypes occupied all sampling locations. With regards to *G*. *glycymeris*, the most common haplotypes (H8 and H10) that were found both in UK and France populations may represent the ancient haplotypes that probably evolved when the English Channel was just a river (Channel River). At that time (ca. 21 000 BP), Britain and Ireland were part of continental Europe, partly covered by the Fennoscandian ice sheet [63]. According to the divergence time estimation applied on mtDNA, the speciation process of *G*. *glycymeris* and *G*. *pilosa* started to occur in the Eocene, during a time of warm climate, while the accelerated intraspecies divergence occurred during the late Pliocene and Pleistocene. During the late Miocene and early Pliocene, the Mediterranean experienced dramatic palaeoceanographic events, a more restricted system with warmer temperatures was delimited with higher salinity and nutrient impoverishment of the marine environment [64–65]. The desiccation during the Messinian Salinity Crisis (MSC) ~5.6–5.3 million years ago caused a major extinction of the Mediterranean marine fauna. A new environmental setting with open-marine conditions (temperate waters and high productivity), with the opening of the Strait of Gibraltar, favored the introduction of species of Atlantic origin, known as Boreal Guests [64,66–67]. Fossil *Glycymeris* sp. shells coming from the Early Pleistocene deposits cropping out in the Arda River section (Castell’Arquato Formation, Italy), have been found to resemble recent specimens of *G*. *glycymeris* from Brittany (France) in shell ultrastructure comparisons. This finding confirms their introduction during this period and suggest that it didn’t change much during the last 2 million years [11], supporting the high conservatism of glycymerids [23]. Other fossil malacofauna confirmed the existence of *G*. *glycymeris* in Pliocene sediments [45,68–69] and during the Pleistocene [10].

The diversity of *Glycymeris* species in the Mediterranean includes *G*. *glycymeris*, *G*. *pilosa*, *G*. *bimaculata* and *G*. *nummaria* (known in the past as *G*. *violacescens* (Lamarck, 1819) and as *G*. *insubrica* (Brocchi, 1814), the latter as a Pliocene fossil). Whereas both *G*. *glycymeris* and *G*. *pilosa* coexist in the western Mediterranean, there is still uncertainty regarding the eastern basin [15] indicating Sicily as a possible geographical barrier for *G*. *glycymeris* [16]. The presence of this or both species in the eastern Mediterranean has led to misleading citations of *G*. *pilosa* as *G*. *glycymeris* throughout time (e.g.,[12,70]) making it unclear whether *G*. *glycymeris* can be found in the eastern Mediterranean Sea. Although the collection of living specimens of *G*. *glycymeris* in the western Mediterranean has not been possible for this study, there is evidence of its presence (recorded captures, collections and personal communications) on the coasts of Spain, France and Italy [16] (Natural History Museum Collection in Milan, R.Chemello pers. comm., S.Giacobbe pers. comm.). The only citation of *G*. *glycymeris* in the eastern Mediterranean corresponds to the Hellenic seas [71]; however, it could not be confirmed. Traditionally, the distribution of shallow marine organisms has been affected by abiotic factors such as salinity and temperature. The field settings of the present study represent two different environments. The Atlantic populations of *G*. *glycymeris* live in deep waters (approx. 20 m) under strong current regimes within sand and occasionally muddy areas [26]. Mediterranean populations of *Glycymeris pilosa*, come from shallower, intermittently calm waters (between 3–6 m), within a sediment layer of coarse skeletal sand with occasionally shell gravel on a limestone platform, characteristic of the Adriatic coast. Their presence in these kinds of habitats may be explained as an advantage to colonize nutrient-limited habitats due to their free-burrowing mobility and it is this ability that makes Glycymerid species opportunistic [23,72]. Alike, these opportunistic animals may appear or disappear from a specific area due to undetermined factors [10], such as fluctuations in food supply [65].

The expansion of *G*. *pilosa* across the entire Mediterranean more progressively than *G*. *glycymeris*, could be explained by a higher adaptive capacity to environmental changes of the first, and a lack of adaptation to unfavorable ecological changes on the latter, which can ultimately cause the extinction or near extinction of a species [63]. This evidence is supported by the potential absence of *G*. *glycymeris* in the Eastern Basin.

The presence of *G*. *pilosa* as the only living species of the Glycymerididae in Israel, surrounded by fossil shells of *G*. *nummaria* [73], supports the evidence that *G*. *pilosa* might be more robust to environmental changes, persisting along the Eastern Mediterranean. This robust nature is reflected in the higher level of intraspecific genetic divergence in *G*. *pilosa* Mediterranean immigrants (COI, 1.1%) compared to the *G*. *glycymeris* from Atlantic (COI, 0.4%). Such a feature has already been recognized in mammals and birds where environmental harshness (lower primary productivity, decreased rainfall and more variable and unpredictable temperatures) is positively correlated with intraspecific divergence [74].

This assumption cannot be confirmed despite our efforts in collecting living specimens of *G*. glycymeris in the Western basin, mainly because the scarce presence of individuals (based on previously cited pers. comm.) and its non-commercial nature that hampers its access at fish markets.

To gain a better understanding in the introduction and expansion of *Glycymeris* in the Mediterranean or even to compile all European glycymerids, further studies on fossil shells to build hypothetical divergent pathways would be very interesting. Additionally, revising existing records in Museum collections is encouraged to identify potential misleading identifications.

The Mediterranean Sea has been assessed for potential impacts on marine biodiversity, indicating the central Adriatic as one of the areas with major cumulative threats to invertebrate species [75]. Since Marine Protected Areas (MPAs) are still scarce in the Mediterranean, delimiting species with functional or evolutionary traits is critical to identify priority areas for marine biodiversity protection [76]. The present study provides the basis for the correct identification of an invertebrate species with high potential for future ecological and sclerochronological studies in an area under high anthropogenic threat.

## Conclusions

The present study resolves the misleading identification of *G*. *pilosa* as *G*. *glycymeris* through the combined use of morphological (classic and landmark-based) and genetic tools. It also sets a guideline for the correct identification of these species. *Glycymeris pilosa* presents a more globose shape and tends to be more asymmetric than *G*. *glycymeris*. Microsculpture provides robust information for the identification of the studied species. The mitochondrial and nuclear genes proved the genetic distance between species; the COI marker appeared to be more informative for the phylogeny of both higher-level and closely related *Glycymeris* species than the ITS2. The molecular clock showed that the ancient clades leading to G. *glycymeris* and *G*. *pilosa* diverged during the Eocene and based on our results, their coexistence in the Mediterranean could have been driven by geological events such as the Messinian Salinity Crisis. Climatic and biotic changes influenced the expansion of both species throughout the Mediterranean favouring *G*. *pilosa*–a more tolerant species and better adapted to unfavorable changes. The outcome of this study sets the baseline for future studies on palaeoenvironmental archives in the Mediterranean.

## Supporting Information

S1 Fig LDA biplot of the previous PCA. Symbols are assigned to each population: *Glycymeris glycymeris* (black inverted triangle, UK; grey inverted triangle, France) and *Glycymeris pilosa* (black circle, Pag; grey circle, Pašman).
(PNG)

S2 Fig Bayesian posterior probabilities and bootstrap support for MrBayes and Maximum Likelihood analyses. Illustration of tree topology based on ITS haplotypes of *G*. *glycymeris* (Gg), *G*. *pilosa* (Gp), *G*. *nummaria* (Gn) and *G*. *bimaculata* (Gb). Posterior probabilities followed by bootstrap values are included at the nodes. The origin of the haplotypes (H) is indicated as follow: UK, United Kindom; FR, France; AD, Adriatic Sea; AT, Atlantic Sea; PA, Pag; PS, Pašman.
(TIF)

S3 Fig Evolutionary time tree of Glycymeridae relationships inferred from Bayesian inference analyses with BEAST of COI gene. Text adjacent to selected nodes indicates median ages. Blue bars indicate 95% highest posterior density intervals for nodes of interest. Text below selected nodes indicates posterior probabilities.
(TIF)

S1 Table Other material of *Glycymeris pilosa* and *Glycymeris glycymeris* from National Museum of Wales collection used for rib count measurements. (DOC)

S2 Table Number of COI and ITS2 haplotypes obtained in each population of *G*. *glycymeris* and *G*. *pilosa*. Pop 1 in *G*. *glycymeris* stands for population sampled in United Kingdom, while Pop 2 stands for population sampled in France. For *G*. *pilosa* Pop 1 was sampled near the Island of Pag and Pop 2 in vicinity of the Island of Pašman, both in the Adriatic Sea.
(DOC)

S3 Table Descriptive statistics of genetic diversity and demographic history of *Glycymeris glycymeris* and *Glycymeris pilosa*, based on COI and ITS2 sequence data. N is number of analysed sequences, h is haplotype diversity (±SD), π is nucleotide diversity (±SD) and S is number of segregating sites.
(DOC)
