# Supplementary figures and images for: Combined Use of Morphological and Molecular Tools to Resolve Species Mis-Identifications in the Bivalvia The Case of Glycymeris glycymeris and G. pilosa
Source: PLoS One. 2016 Sep 26;11(9):e0162059. doi: 10.1371/journal.pone.0162059 (PMC5036790; doi:10.1371/journal.pone.0162059)

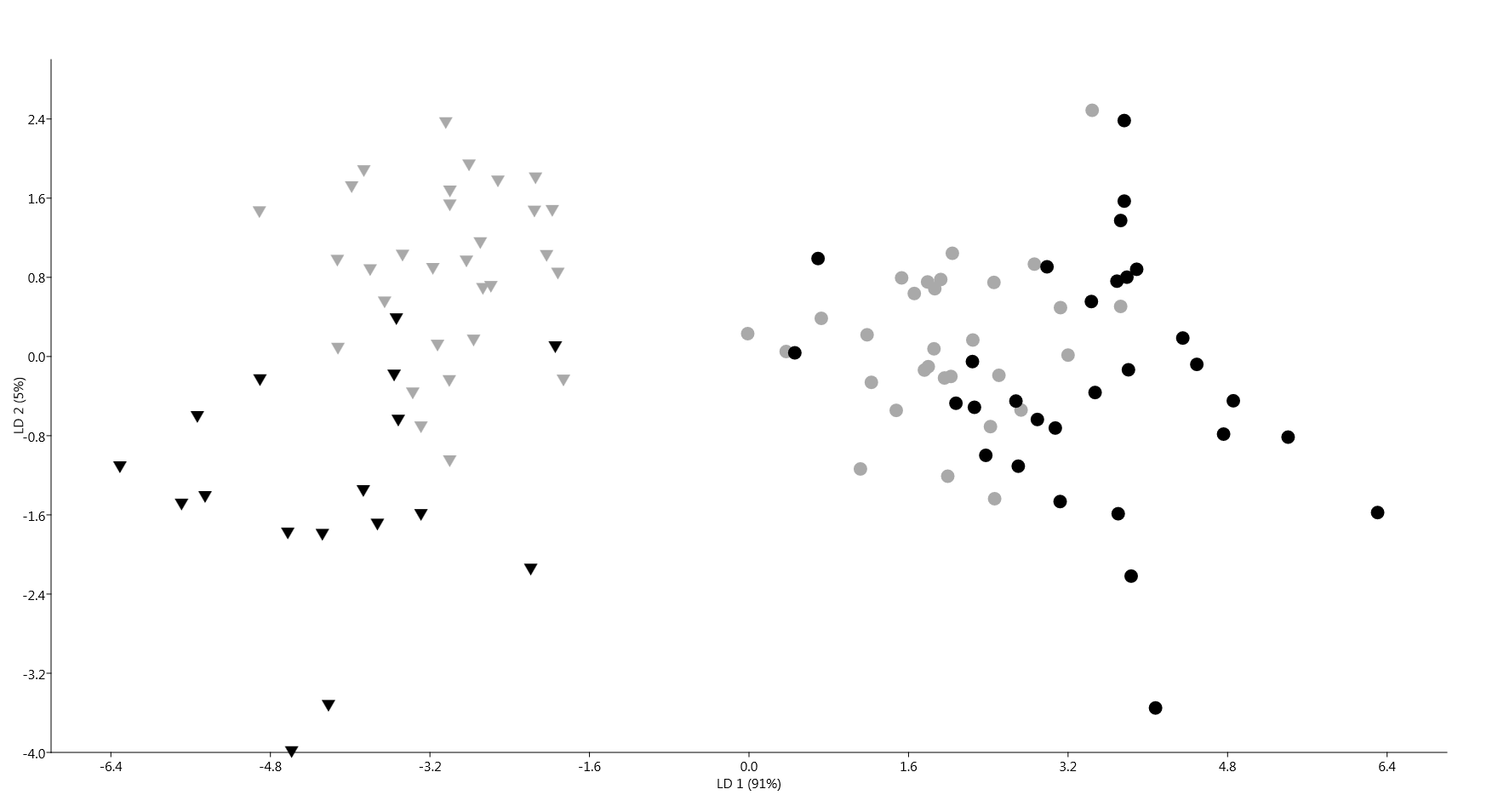

Supplement: S1 Fig — Symbols are assigned to each population: Glycymeris glycymeris (black inverted triangle, UK; grey inverted triangle, France) and Glycymeris pilosa (black circle, Pag; grey circle, Pašman). (PNG) [file pone.0162059.s001.png]

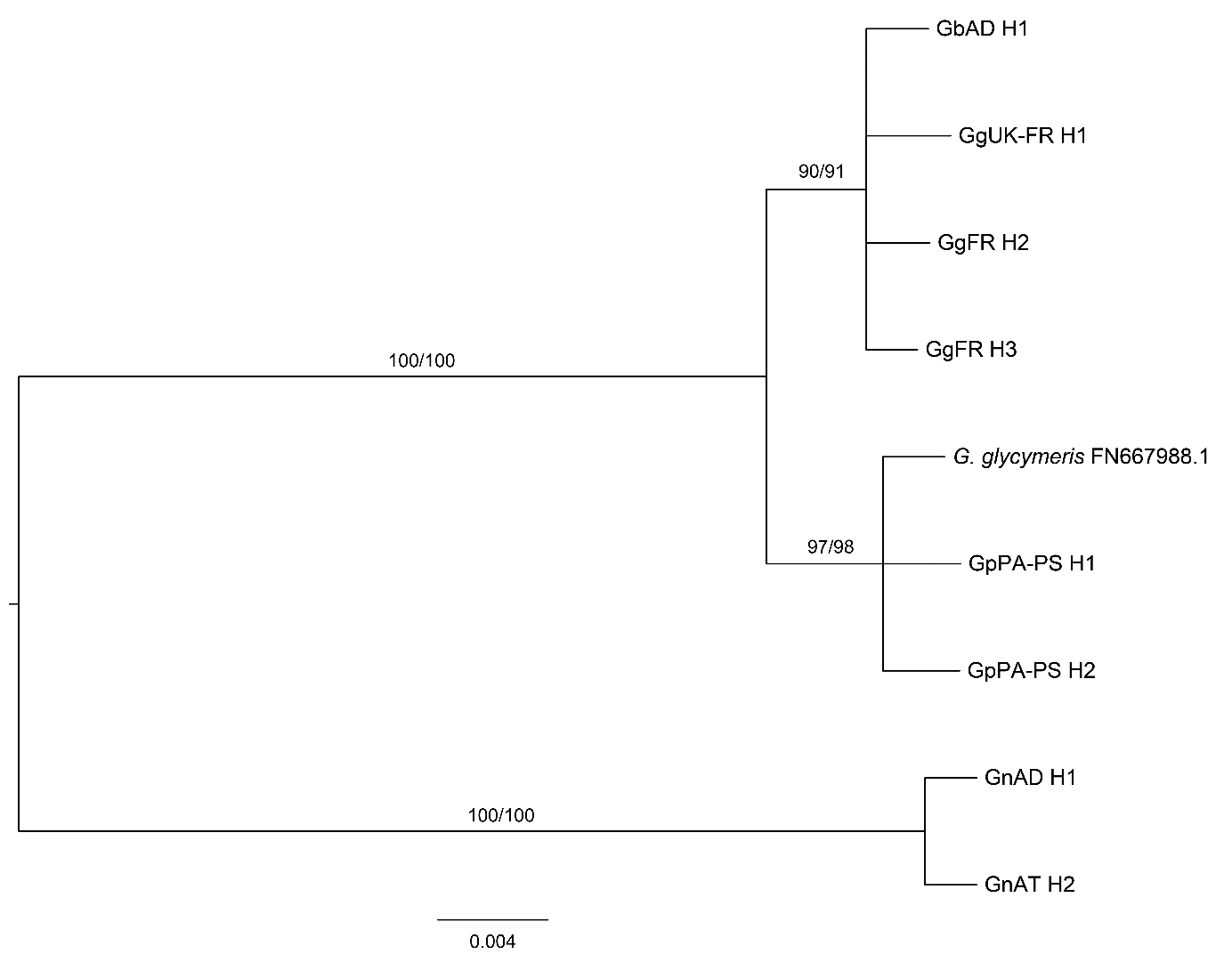

Supplement: S2 Fig — Illustration of tree topology based on ITS haplotypes of G. glycymeris (Gg), G. pilosa (Gp), G. nummaria (Gn) and G. bimaculata (Gb). Posterior probabilities followed by bootstrap values are included at the nodes. The origin of the haplotypes (H) is indicated as follow: UK, United Kindom; FR, France; AD, Adriatic Sea; AT, Atlantic Sea; PA, Pag; PS, Pašman. (TIF) [file pone.0162059.s002.tif]

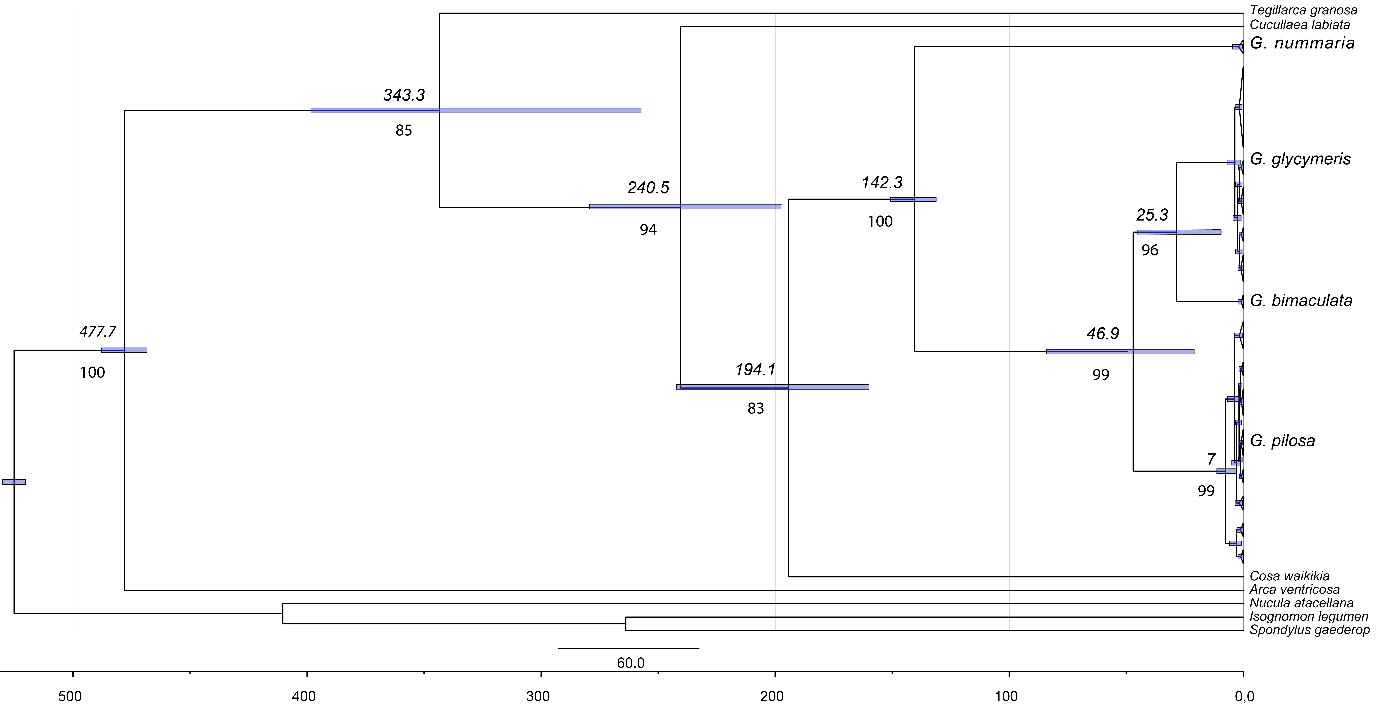

Supplement: S3 Fig — Text adjacent to selected nodes indicates median ages. Blue bars indicate 95% highest posterior density intervals for nodes of interest. Text below selected nodes indicates posterior probabilities. (TIF) [file pone.0162059.s003.tif]
