# Supplementary material for: Combined Use of Morphological and Molecular Tools to Resolve Species Mis-Identifications in the Bivalvia The Case of Glycymeris glycymeris and G. pilosa
Source: PLoS One. 2016 Sep 26;11(9):e0162059. doi: 10.1371/journal.pone.0162059 (PMC5036790; doi:10.1371/journal.pone.0162059)
Supplement: S1 Table — (DOC) [file pone.0162059.s004.doc]

**S2 Table.** Other material of *Glycymeris pilosa* and *Glycymeris glycymeris* from National Museum of Wales collection used for rib count measurements.

| **Location** | **Secondary rib count per 5mm** | **Species** |
| --- | --- | --- |
| 1) *Glycymeris pilosa,* Cetina River, Croatia, Mediterranean, 43°26’13”N 16°41’14”E collected by M.Peharda Uljevic 6/5/14 ID: CG0/1. NMW.Z.2014.023.00003. | 12 | *Glycymeris pilosa*  *12-18* |
| 2) *Glycymeris pilosa,* Pag Bay, North Dalmatia, Croatia, Mediterranean, 44°27’42”N 15°01’36”E collected by M.Peharda Uljevic 3/5/14 ID: AG0/15. NMW.Z.2014.023.00002. | 12 |
| 3) *Glycymeris pilosa,* Pašman Channel, Croatia, Mediterranean, 43°56’49”N 15°23’18”E collected by M.Peharda Uljevic 8/5/14 ID: BG0/3. NMW.Z.2014.023.00001. | 14 |
| 4) *Glycymeris pilosa*, Pašman Channel, Croatia, Mediterranean, 43°56’49”N 15°23’18”E collected by A.Purroy Albet 2/8/15 ID: BG15/1. IOF. | 12 |
| 5) *Glycymeris pilosa,* Istria, Croatia, Mediterranean, 45°15’14.6”N 13°35’19”E collected by M.Peharda Uljevic 12/5/14 ID: 3. NMW.Z.2014.023.00004. | 15 |
| 6) *Glycymeris glycymeris*, Dorset, UK, NE Atlantic, collected by I.Killeen 25/6/88. NMW.Z.2009.036.03091. | 23 | *Glycymeris glycymeris*  *14-28* |
| 7) *Glycymeris glycymeris,* Dorset, UK, NE Atlantic, collected by I.Killeen 25/6/88. NMW.Z.2009.036.03091. | 24 |
| 8) *Glycymeris glycymeris,* Isle of Man, UK, NE Atlantic, 54°26’54.49’’N 4°20’21.73’’W collected by I.Bloor 9-13/4/15. IOF. | 23 |
| 9) *Glycymeris glycymeris* Fish market, St Pol de Leon, France 5/1997. NMW.Z.2009.036.03082. | 21 |
| 10) *Glycymeris glycymeris* Fish market, St Pol de Leon, France 5/1997. NMW.Z.2009.036.03082. | 22 |
| 11) *Glycymeris glycymeris* Pointe du Chevet, Ile de la Colombiere, Brittany, France, NE Atlantic, 48°37.8’N 02°11.9’W collected by I.Killeen 29/3/98. NMW.Z.2009.036.03092. | 24 |
| 12) *Glycymeris glycymeris,* Pointe du Chevet, Ile de la Colombiere, Brittany, France, NE Atlantic, 48°37.8’N 02°11.9’W collected by I.Killeen 29/3/98. NMW.Z.2009.036.03092. | 27 |
| 13) *Glycymeris glycymeris,* Pointe du Chevet, Ile de la Colombiere, Brittany, France, NE Atlantic, 48°37.8’N 02°11.9’W collected by I.Killeen 29/3/98. NMW.Z.2009.036.03092. | 27 |
| 14) *Glycymeris glycymeris,* Dinard, Ille-et-Vilaine, Brittany, France, NE Atlantic, 48°37.8’N 02°11.9’W collected by I.Killeen 29/3/98 2009.036.03092. NMW.Z.2009.036.03092. | 20 |
| 15) *Glycymeris glycymeris* Dinard, Ille-et-Vilaine, Brittany, France, NE Atlantic, 48°37.8’N 02°11.9’W collected by I.Killeen, 29/3/98 2009.036.03092. NMW.Z.2009.036.03092. | 22 |
| 16) *Glycymeris glycymeris* Dinard, Ille-et-Vilaine, Brittany, France, NE Atlantic, 48°37.8’N 02°11.9’W collected by I.Killeen 29/3/98 2009.036.03092. NMW.Z.2009.036.03092. | 28 |
| 17) *Glycymeris glycymeris,* Bay of Brest, France, NE Atlantic, 48°20’29’’N 4°30’46’’W, collected by A.Featherstone 9/3/15 15031 017. IOF. | 20 |
| 18) *Glycymeris glycymeris,* Bay of Brest, France, NE Atlantic, 48°20’29’’N 4°30’46’’W, collected by A.Featherstone 9/3/15 15031 002. IOF. | 18 |
