# Supplementary material for: Combined Use of Morphological and Molecular Tools to Resolve Species Mis-Identifications in the Bivalvia The Case of Glycymeris glycymeris and G. pilosa
Source: PLoS One. 2016 Sep 26;11(9):e0162059. doi: 10.1371/journal.pone.0162059 (PMC5036790; doi:10.1371/journal.pone.0162059)
Supplement: S2 Table — Pop 1 in G. glycymeris stands for population sampled in United Kingdom, while Pop 2 stands for population sampled in France. For G. pilosa Pop 1 was sampled near the Island of Pag and Pop 2 in vicinity of the Island of Pašman, both in the Adriatic Sea. (DOC) [file pone.0162059.s005.doc]

**S4 Table. Number of COI and ITS2 haplotypes obtained in each population of *G. glycymeris* and *G. pilosa.***

|  | *Glycymeris glycymeris* | |  | *Glycymeris pilosa* | |
| --- | --- | --- | --- | --- | --- |
| COI |  |  | COI |  |  |
| Haplotypes | Pop 1 | Pop 2 | Haplotypes | Pop 1 | Pop 2 |
| Hap1 |  | 1 | Hap1 | 1 | 1 |
| Hap2 |  | 1 | Hap2 | 1 |  |
| Hap3 |  | 3 | Hap3 | 3 | 1 |
| Hap4 |  | 1 | Hap4 | 1 |  |
| Hap5 |  | 1 | Hap5 | 1 |  |
| Hap6 |  | 1 | Hap6 | 1 |  |
| Hap7 |  | 1 | Hap7 | 1 |  |
| Hap8 | 5 | 1 | Hap8 | 1 |  |
| Hap9 |  | 1 | Hap9 | 1 |  |
| Hap10 | 6 | 3 | Hap10 | 1 |  |
| Hap11 |  | 1 | Hap11 | 1 |  |
| Hap12 |  | 1 | Hap12 | 1 | 5 |
| Hap13 | 1 |  | Hap13 | 1 | 1 |
| Hap14 | 1 |  | Hap14 |  | 2 |
| Hap15 | 1 |  | Hap15 |  | 1 |
| Hap16 | 1 |  | Hap16 |  | 1 |
|  |  |  | Hap17 |  | 1 |
|  |  |  | Hap18 |  | 1 |
|  |  |  | Hap19 |  | 1 |
| total | 15 | 16 | total | 15 | 15 |
| ITS2 |  |  | ITS2 |  |  |
| Haplotypes | Pop 1 | Pop 2 | Haplotypes | Pop 1 | Pop 2 |
| Hap1 | 15 | 13 | Hap1 | 11 | 12 |
| Hap2 |  | 1 | Hap2 | 3 | 3 |
| Hap3 |  | 1 |  |  |  |
| total | 15 | 15 | total | 14 | 15 |

Pop 1 in *G. glycymeris* stands for population sampled in United Kingdom, while Pop 2 stands for population sampled in France. For *G. pilosa* Pop 1 was sampled near the Island of Pag and Pop 2 in vicinity of the Island of Pašman, both in the Adriatic Sea.
