# Supplementary material for: Combined Use of Morphological and Molecular Tools to Resolve Species Mis-Identifications in the Bivalvia The Case of Glycymeris glycymeris and G. pilosa
Source: PLoS One. 2016 Sep 26;11(9):e0162059. doi: 10.1371/journal.pone.0162059 (PMC5036790; doi:10.1371/journal.pone.0162059)
Supplement: S3 Table — N is number of analysed sequences, h is haplotype diversity (±SD), π is nucleotide diversity (±SD) and S is number of segregating sites. (DOC) [file pone.0162059.s006.doc]

**S5 Table. Descriptive statistics of genetic diversity and demographic history of *Glycymeris glycymeris* and *Glycymeris pilosa*, based on COI and ITS2 sequence data.**

| Region | Bivalve species | N | h (±SD) | π (±SD) | S | Tajima’s D | Fu’s F_ST_ |
| --- | --- | --- | --- | --- | --- | --- | --- |
| COI | *G. glycymeris* | 31 | 0.884±0.042 | 0.0030±0.0004 | 15 | -1.55  (p=0.05) | -11.61  (p=0.00) |
|  | *G. pilosa* | 30 | 0.945±0.027 | 0.0085±0.0016 | 32 | -1.07  (P=0.13) | -6.98  (p=0.01) |
| ITS2 | *G. glycymeris* | 30 | 0.131±0.082 | 0.0004±0.0003 | 2 |  |  |
|  | *G. pilosa* | 29 | 0.340±0.090 | 0.0011±0.0003 | 1 |  |  |

N is the number of analysed sequences, h is haplotype diversity (±SD), π is nucleotide diversity (±SD) and S is the number of segregating sites. Tajima’s D and Fu’s F_ST_ are the two neutrality tests applied.
